# Examining the health and wellness of solo self-employed workers through narratives of precarity: a qualitative study

**DOI:** 10.1186/s12889-024-18179-5

**Published:** 2024-03-06

**Authors:** Tauhid Hossain Khan, Ellen MacEachen

**Affiliations:** 1 School of Public Health Sciences, University of Waterloo, Waterloo, ON Canada; 2 Department of Sociology, Jagannath University, Dhaka, Bangladesh

**Keywords:** Future work, Health, Wellness, Precarity, Precariat class, Solo Self-Employment, Independent contractor, Neoliberalism

## Abstract

**Background:**

In recent decades, there has been a significant transformation in the world of work that is characterized by a shift from traditional manufacturing and managerial capitalism, which offered stable full-time employment, to new forms of entrepreneurial capitalism. This new paradigm involves various forms of insecure, contingent, and non-standard work arrangements. Within this context, there has been a noticeable rise in Self-Employed individuals, exhibiting a wide range of -working arrangements. Despite numerous investigations into the factors driving individuals towards Self-Employment and the associated uncertainties and insecurities impacting their lives and job prospects, studies have specifically delved into the connection between the precarious identity of Self-Employed workers and their overall health and well-being. This exploratory study drew on a ‘precarity’ lens to make contributions to knowledge about Self-Employed workers, aiming to explore how their vulnerable social position might have detrimental effects on their health and well-being.

**Methods:**

Drawing on in-depth interviews with 24 solo Self-Employed people in Ontario (January – July 2021), narrative thematic analysis was conducted based on participants' narratives of their work experiences. The dataset was analyzed with the support of NVIVO qualitative data analysis software to elicit narratives and themes.

**Findings:**

The findings showed that people opt into Self-Employment because they prefer flexibility and autonomy in their working life. However, moving forward, in the guise of flexibility, they encounter a life of precarity, in terms of job unsustainability, uncertainties, insecurities, unstable working hours and income, and exclusion from social benefits. As a result, the health and well-being of Self-Employed workers are adversely affected by anger, anomie, and anxiety, bringing forward potential risks for a growing population.

**Conclusion and implications:**

Neoliberalism fabricates a ‘precariat’ Self-Employed class. This is a social position that is vague, volatile, and contingent, that foreshadows potential threats of the health and wellbeing of a growing population in the changing workforce. The findings in this research facilitate some policy implications and practices at the federal or provincial government level to better support the health and wellbeing of SE'd workers.

## Background

Prodigious global socio-economic and cultural forces were pointed out by David Harvey in the 1970s, when he noted, “There has been a sea-change in cultural as well as in political-economic practices since around 1972” [1]. These social changes are historical; societies changed from agrarian to industrial (e.g. manufacturing-based economy) before embarking onto the current post-industrial society (e.g., service-based economy) [2]. As part of this social transformation, work relationships and arrangements have changed in recent decades, as part of a 'paradigm shift' from manufacturing/managerial capitalism with full-time, secure, and standard employment relationships to entrepreneurial capitalism, with precarious, contingent, and non-standard working arrangements [3, 4]. These transformations have impacted many aspect of human lives, including occupational relations and patterns. This historical transition escalated the destandardization of work and prompted changes to three key dimensions of standard working relations: contract type (e.g. SE, gig work), spatial dimensions (e.g. homeworking), and temporal dimensions (e.g. temporary/part-time work) [5, 6]. This transition also created the alleged ‘flexibility’ of work as a double-edged sword for workers. On the one hand, flexible employment helped work-life balance; on the other hand, it elevated employment insecurity and vulnerability as workers increasingly fell outside of coverage of social security support systems [7–9]. Although people’s health and wellbeing are important to state economies, how these labour market /social transformations affect people’s health and wellbeing has been surprisingly minimally addressed.

In Western societies, understandings of the impacts of changing social structures on human lives during industrialisation were mainly fuelled by three leading classical sociologists, who paid attention to how changing working life affects populations’ health and wellbeing. Durkheim identified an ‘anomic’ condition [10], Marx stressed ‘alienation’ (psychological or social illness) [11], and Weber analyzed how people were caught in an ‘iron cage’ by bureaucratic organization [12]. In each instance, the theorists described health in relation to the new form of social structure, including work arrangements. In a similar vein, later sociologist Talcott Parsons described the “sick role” in 1951 and Merton described “unintended consequences” in 1936 as new ways to understand human lives and society as a whole [13, 14]. In the last few decades, prominent sociologists have moved on from these theories to place the concept of ‘precarity’ at the center of their analysis of social transformation to explain the meaning and origins of precarious life and work.

The ‘labour market question’ has received significant attention from contemporary sociologists, who have described new forms of the labour market and a new class of working people. Giddens (1991) wrote of “ontological insecurity” as a defining feature of contemporary social life and as the outcome of what he calls “reflexive modernization”[12]. Beck (1992, 2000) proposed that the side effects of reckless economic growth have led to the emergence of the “risk society,” in which scientific and technological expertise multiply the threats that people face daily. Both Beck and Castells portrayed the processes of work de-standardization as evolving. Beck (2000) focused on conceptions of non-standard work, such as part-time work, inconsequential and temporary employment, and spurious forms of SE; while Castells (2011) stressed part-time work, temporary work, and SE. Their analyses often overlap [9]: Beck (2000) refers to the work ‘revolution’ in relation to lean production, subcontracting, outsourcing, offshoring, downsizing, and customization, and Castells (2011) refers to the transformation of work and how the emergence of lean production methods go hand in hand with the widespread business practice of subcontracting, offshoring, downsizing, and customizing [9]. As a result, insecure terms of employment and flexible work are developing faster than any other type of work due to competition-induced, technology-driven trends [8, 9]. Thus, according to Beck (1992), risks have been transferred from institutions to individuals, and these processes involve the development of a risk-fraught system of flexible, pluralized, decentralized, underemployment. Beck saw this elevated insecurity as spreading globally, linking work to poverty, and creating a new ‘working poor’ in non-standard labor markets [8]. Castells (2011) outlined the evolving workforce as marked by diverse flexibility in both worker roles and working circumstances, regardless of skill levels. Bauman (2000) observed an emerging era characterized by the dissolution of solid, established institutional frameworks that supported industrial capitalism, ushering in a phase of "liquid modernity." Within this context, the prevailing essence of contemporary life involves widespread and deeply felt precariousness, instability, and vulnerability (Bauman, 2000, pp. 160–161). Harvey (1991) portrayed the labor market transition from Fordism to flexible accumulation in a context of political-economic transformation, resulting in restructuring the labor market into more flexible forms, cutting away at traditionally well-compensated core jobs.At the same time, a shift in employment from manufacturing to service work has accelerated. Harvey identifies four major increases in corporate business trends during the current postmodern era, which began in the 1970s and took hold in the 1990s: (1) mergers; (2) corporate diversification; (3) self-employment; and (4) outsourcing. Harvey suggests that the sense of life as new, fleeting, ephemeral within postmodernity maps onto the characteristics of capital flows during the postmodern period.

This elevated insecurity profoundly affects workers’ ability to change or improvise their labour market position. For example, Bourdieu argued that this labor market uncertainty weakens the possibility of engaging in collective action [15, 16].

Altogether, the expansion of precarious work aligns closely with a system of control and exploitation that subtly wields social and political authority over an elevated wide portion of the workforce. Bourdieu identifies the institutionalization of precarious work as a potential catalyst for the emergence of "a mode of domination of a new kind” (1998, p.85). This viewpoint finds resonance in the thoughts of philosopher Judith Butler. While Butler initially asserted (2004) that post-9/11 society had normalized violent conflicts and warfare, she [17, 18] later deduced that precarious economic circumstances represent "not a transient or sporadic state, but a new mode of regulation that defines this historical era" (Butler, 2015, p. vii [19]). She frames precarity as an established regime, a prevailing method of governance for both society and self. A central theme evident across a significant portion of this literature is that the process of labor becoming part of the precariat has assumed a political role, fostering a heightened state of compliance not merely despite, but specifically due to, the uncertainties introduced by neoliberalism. This neoliberal policy engendered a new and sophisticated type of exploitation of labour resources, which relieves the employers of responsibility for the normal existence of millions of people and their families. As such, this new social structure has been described as a new class: the working poor or precariat class [20]. SE is a part of precarious work that has been growing rapidly with different contours and configurations in recent decades due to above-mentioned forces of social and labour market changes [21–24].

While various studies have observed the reasons behind the people entering into SE and related uncertainty and insecurity related to life and job sustainability, and have discussed how SE’d workers are left outside of government support systems [23, 25, 26], none specifically have looked at the relationships between SE as precariat class identity and their health and wellbeing. While some studies have examined relationships between SE/precarious employment and health, these are based on narrow depictions of specific variables [27–30].The purpose of this study is not to quantify or measure the association, the depth and breadth of health and wellbeing of SE’d workers, nor to provide an account of their vulnerabilities or marginalized position in the labor market. Rather it is an exploratory study that draws on a precarity lens, aiming to examine how precarity affects the health and wellbeing of SE’d workers.

### Self-employed workers: a precariat class

As was mentioned earlier precarious and nonstandard work has been growing rapidly globally [21–24]. The International Labour Organization estimated that non-standard employment accounted for more than 60% of workers worldwide in 2015, and the percentage would be higher now [31]. Overall, SE’d workers make up approximately 15% of the workforce in Canada [32], 10% of the Australian workforce [33], and 15% of the workforce in Europe [29]. The emergence of the 'gig economy' has played a significant role in the current trend of the increasing prevalence of SE. This is also fueled by the decline of conventional employment models that used to offer stable income and lifelong job security [6, 22, 34–36].

SE'd workers have frequently been portrayed as a homogenous cohort comprising individuals who experience sound health, relish the independence of self-employment, benefit from flexible work schedules, and do not depend on government social protections. This working population has been depicted as experiencing elevated levels of job satisfaction, an enhanced quality of life, and improved prospects for achieving a harmonious work-life balance compared to traditional employees [29, 37–39].

SE’d individuals have also had a reputation for taking on substantial personal risk to establish their own ventures and generate employment opportunities for others [26, 29, 34, 40]. Nevertheless, these portrayals have more recently come to represent just one facet of the labor market landscape, as a considerable portion of self-employed (SE'd) workers find themselves compelled to embrace this employment arrangement due to factors such as joblessness, limited alternatives, and financial constraints [26, 29, 41–46]. In a contrast to depictions of SE’d as homogenous, the diversity of SE’d workers was described by the Law Commission of Ontario (2012), which noted that: “the experiences and vulnerabilities of this group range from billionaire entrepreneurs to taxi drivers working 90 h a week simply to pay their bills and includes many people who are gaining income from SE activity alongside their main job” (LCO, 2012: 75).

Therefore, SE does not always mean health and self-sufficiency. Instead, some SE’d workers may be considered as precarious workers at risk of poverty and social exclusion [47] because they have low job and income security, poor working conditions, and low social security coverage [22, 47, 48]. Of importance is that, globally, SE’d workers are largely excluded from formal support systems such as workers’ compensation, employment insurance, and state pension plans [22, 23]. Consequently, in Canada, Australia, and other regions, a growing number of self-employed workers face low wages and struggle to cover essential expenses such as housing, medical bills, and food. The absence of social safety nets further compounds their concerns about securing their future, including aspects like retirement pensions. This study questions whether SE’d workers need income support during their withdrawal from work due to injury or sickness [49].

### Impact of precarity on health and wellbeing

Increasing international evidence highlights that the rise of precarious self-employment is significantly impacting workers' safety, health, and overall well-being in detrimental ways [4, 26, 30, 36]. While self-employment is not a recent form of work, advancements in communication and information technologies have expanded its scope [23, 24]. Studies have demonstrated that employment precarity leads to a multitude of insecurities and uncertainties in workers' lives, affecting their income, job sustainability, family dynamics, and social lives [4, 50–53]. An increasing body of evidence has found that pressures stemming from insecure work and income have significant consequences for individuals' health, well-being, and illness, and are closely linked to adverse outcomes in both mental and physical health [26, 28, 52]. Apart from the health impacts of precarious work on self-employed workers, various studies have shed light on the physical and mental health risks prevalent in certain self-employed sectors. Notably, in industries like food and farming, self-employed workers face a heightened risk of specific diseases including musculoskeletal disorders, joint pain, sleep disturbances, and digestive complaints, and surpassing the risks encountered by salaried workers in the same sectors [26, 54]. Although some studies have reported SE’d workers to be healthier than salaried workers [26, 55–57], these health differences between regular employees and SE’d workers can be explained by the ‘selection effect’ [26]. That is, these studies could be affected by the 'healthy worker effect,' wherein healthier workers are the subject of investigation or individuals with better health might choose self-employment over other forms of work, leading to a bias in the findings [26]. Given the growing portion of low-income SE’d, it is our view that SE can have a considerable adverse impact on workers’ health and personal lives, including family relations [30, 54, 58].

### Theoretical framework: neoliberalism and precariat class

This section presents the two axes of theoretical perspectives to understand the precariatization of the labor market. One axis relies on the perspective of political economy, delineating how neoliberal ideas breed a precarious labor market; the second refers to the work of contemporary sociologists, mentioned in the background section, who brought the concept of ‘precarity’ into discussion of modernity/late modernity/late capitalism/postmodernity. Guy Standing, building on these two macro perspectives, developed the idea of a ‘precariat’ or a ‘New Dangerous Class’ in the context of the current labor market, and as different from the era of the Fordist employment regime with its stable, secure employment.

Historically, the ‘precariat’ as a class, or precariatization of labour market, is the byproduct of neoliberalism, which emerged starting in the 1960s and 1970s [50]. David Harvey (2005), one of the pioneers who identified the traits of neoliberalism, while inspired by Marxist thoughts, stressed that neoliberalism is a political and economic project aiming at restoring the monopoly of capitalist hegemony over states by limiting the power of working classes and involving privatization, deregulation, and intervening macroeconomic policies. He also argued that neoliberal policies uplift material interests (e.g., income and wealth) of capitalists at the expense of deteriorating living conditions for the poor and working classes [59]. Thus, neoliberal shifts in states across the world made people more dependent on market mechanisms and less dependent on states. Essentially, neoliberal markets have dismantled and reversed advancements of welfare states [59]. Neoliberal discourse posits that social guarantees for the working class and concessions to labor unions inevitably slowed economic growth, accelerated de-industrialization, and undercut production efficiency [59]. Consequently, governments and states curtailed social benefits to ensure market supremacy. Essentially, an important aim of neoliberalism is to shift the burden of risks and concerns about social and personal life onto individuals themselves [59]. Harvey (2005) notes that the implementation of neoliberal ideas did, in some ways, make the economy more efficient, but, at the same time, it led to a distortion of the social structure including unemployment, and the emergence of social groups whose positions were vague, unstable, and ambiguous.

The stratum that emerged was fast becoming a social class, and began to be referred to for the first time as the’ precariat.’ The word “precariat” stems from a combination of two words: the Latin ‘precarium,’ which means unstable or not guaranteed, and ‘proletariat,’ which refers to a socioeconomic class that is cut off from the output of labour and exploited by the ruling class [52]. Against this nexus, Guy Standing prompted us to think back to Karl Marx’s two antagonistic classes: proletariat and bourgeoisie, but within post-industrial neoliberal, capitalistic society. Standing named the precariat class a ‘New Dangerous Class’ [51]. In the evolution of the labour market, the mobilization of the ‘old’ proletariat was followed by the white-collar ‘salariat’ workers. The salariat are those employees who, according to Standing, have stable full-time jobs with benefits such as pensions, paid holidays, and enterprise benefits [51]. However, the salariat is found almost always in large corporations, government agencies, public administration, and the civil service [51]. These classifications are important because Standing argues that the precariat is expanding. He pinpoints a series of changing phenomena that lead to the casualization of employment, including the commodification of the firm, numerical and functional flexibility, job insecurity, occupational dismantling, and wage-system restructuring.

Thus, the historical evolution of employment now gives us the ‘precariat,’ which includes a significant social stratum occupied by members who have precarious socioeconomic conditions and ‘truncated social status,’ such as workers who are temporary, self-employed, part-time, causal, and who are working poor [51]. Despite diverse lifestyles, groups within this social class have some common traits. According to Standing, the precariat is composed of ‘denizens’ who lack key citizenship rights, including social security protections. They also lack key forms of labor-related security, including secure jobs, stable employment, collective representation, decent income, skills with current technologies, and a secure work-based identity that enables people to construct career narratives [51]. Standing indicated that women, young and old people, the less educated, and migrants are most likely to belong to this group [51].The precariat is also disempowered. Both the proletariat of former Marxist theory and the salariat of Standings current theorisation had the advantage of a certain stability of employment, but this stability is denied to the new precariat. Even precariat who perform white-collar work can have precarious incomes as they never know when they are going to be dismissed.

Members of the precariat have a precarious social position leading to the “deintellectualization of labour” and distortion of the labour process. These processes have critical repercussions on today’s young people [52]. The ranks of unemployed are swelled by young people graduating from educational establishments. Temporary or part-time work is a clear sign of a worker’s vulnerability, which neoliberals justify by the urgent need to use labour resources in a flexible manner [52].

Standing sees the precariat as a new class ‘in the making’ in so far as more and more employees find themselves working, as in ‘remunerated’, but not enjoying the fruits of having a ‘position’ they can call theirs within an organisation of which they are a structural part [51]. A job has become a ‘role,’ not a position [51]. A role is ‘’played’, not fixed, and can be played by one person or another. It cannot be ‘held’ nor ‘occupied’ as a solid jobs [51].

The precariat is essentially ‘deprofessionalized’ because they change jobs frequently, not because they want to, but because it is an arrangement imposed by the neoliberal economy on masses of people who increasingly have to work in areas other than those for which they were trained. When they lose their job, they usually get a job in a different sphere that requires a non-specialized background and set of work skills. For example, most of the gig work performed by university students/graduates does not require higher degrees. This deprofessionalization incurs a loss of professional identity and professional culture.

To sum up, the precariat is a new coinage denoting the social stratum that embodies alienation not only among workers from the results of their labour, but from other significant social groups. The members of this group are exposed to particularly sophisticated forms of exploitation of their labor, knowledge, and skills and, ultimately, and of their quality of life. These groups include people who are constantly engaged in temporary and sporadic jobs, owing to which they have truncated social rights and inferior social status.

## Methodology

### Study design

A qualitative methodological approach was utilized for this study due to our interest in how SE'd workers' health and wellness was affected by employment precarity. An interpretative paradigm, which focuses on the understanding of phenomena through meanings people bring to them, was used to reflect upon the narratives provided by participants [60, 61]. This approach helped to unpack the underlying meanings embedded in SE’d workers’ stories, including how everyday practices and experiences are situated in larger structural contexts (e.g., neoliberal market system, precariat class system, social security system). The study was approved by the Research Ethics Board of the University of Waterloo, Canada.

### Participants, sampling, and recruitment

Participants were selected for this study based on the following inclusion criteria: independent contractors with no employees (i.e. solo self-employed), aged 18 years or older, having had experience of illness or injury (work-related or not) while SE’d, main income is from self-employment, working in Ontario (Canada), and (due to researcher language limitations) fluent in English (Table 1). Various social media platforms were used to recruit participants, including Linkedin, Facebook, Kijiji, Twitter, and Tumblr**.** From among eligible participants, we selected participants purposively for information-rich and heterogeneous cases (Patton, 2001). Our final sample was between 21 and 62 years of age, with varied education (college diplomas, university degrees, etc.) and income levels ($25 k/year—$200 k/year). A similar proportion of men and women were included in the study. The lead author interviewed 24 participants using audio/video conferencing with Zoom and WhatsApp. The interviews were conducted between January and July 2021 and lasted 1.10 h on average.

**Table 1:** Participant characteristics

Pseudonym	Gender	Age	Education	Type of SE’d work	Type of illness/injury	F. Income (CAD)/Year
1.Habibur	M	22	College diploma	Uber Driver	Depression Leg fracture	50 K
2.Tasmina	F	32	College diploma	Home childcare	Flu/ fever	50 K
3.Emma	F	36	Undergraduate degree	Catering	Pneumonia	25 K-50 K
4.Mamun	M	45	Graduate degree	Information technology consultant	Spinal Injury	45 K
5.Zayan	M	22	College diploma	Food delivery: Door dash Skip dish	Breaking ankle	100 K
6.Ruby	F	42–47	Graduate degree	Rotary Public commissioner	Depression Stress, Obesity	25 K-50 K
7.Patrick	M	62	Undergraduate degree	Actor, catering	Knee injury	50 K-100 K
8.Sarah	F	54	Graduate degree	Property manager	Stomach pain	50 K-100 K
9.Sumon	M	22	College diploma	Food Delivery	Breaking right hand	25 K-50 K
10.Mary	F	46	High school	Fashion design	Significant autoimmune disorder	< 25 K
11.Faria	F	21	Undergraduate degree	Beautician	ADHD	25 K-50 K
12.Remi	F	45	College diploma	Financial Advisor	Asthma, Covid-19	50 K-10 K
13.Sarika	F	50	High school	Cleaner	Sleep disorder	25 K-50 K
14.Scott	M	50	College diploma	Construction	Arthritis	50 K-100 K
15.Ander	M	25	Postgraduate diploma	Online business/ E-commerce	Anxiety, stress, depression	25 K-50 K
16. Bob	M	33	College diploma	Singer, DJ	Anxiety, stress Back pain	25 K-50 K
17.Jane	F	33	Undergraduate degree	Actor, Writer	Nervous system disorder	130 K
18.Jimmy	M	35	Graduate degree	Data analyst	Regular migraines	200 K
19. Paul	M	32	College diploma	Electrician	Backbone Injury	50 K
20. Ayla	F	35	College diploma	Grocery business	Cardiology ADHD	50 K-100 K
21.Miller	M	24	Undergraduate degree	Music trainer, musician	Leg injury	50 K
22.Mila	F	35	Graduate degree	Tailoring	Backpain, Fatigue	50 K-100 K
23.Arnob	M	30	Graduate degree	Debate /public speaking trainer	Anxiety, stress, burn injury, depression,	25 K-50 K
24.Pablo	F	26	College diploma	Financial advisor	Stress	25 K-50 K

### Data collection

As this study involved soliciting solo SE’d workers’ personal experiences, including culturally sensitive information (e.g., income, sickness, personal family lives), an in-depth interview approach was selected to allow time and space for each person to explain their situation. A semi-structured interview guide was used (Table 2), which was informed by literature and discussion with the research team. We used a combination of questions and probes (follow-up questions) to achieve breadth of coverage across the following key topics: (a) work-related experiences; (b) illness, injury or income reduction/loss, government and informal social benefit systems used; (c) health and wellbeing in the context of work. Interviews were audio-recorded and transcribed verbatim by two professional transcriptionists. Along with a reflexive journal, detailed field notes were taken after each interview to describe encounters, including the immediate impressions and context, and analytic insights.

**Table 2:** Interview Question Domains (created by authors)

1	What type of work you are doing now? Tell me about your work history (chronological)
2	Tell me how you became SE’d?
3	Why did you choose self-employment type work?
4	Can you tell me what types of experience of physical and mental health issues that have impacted your work as a SE’d person, or how your work has affected your overall health and wellbeing?
5	What formal or informal support systems have you used to manage your health and wellness when you faced an illness or injury and were unable to work? By supports, I mean any income, emotional support or help from family, friends, or community members as well as government agencies

### Data analysis: narrative thematic analytical approach

Following Reissman’s (2008) Narrative Thematic Analytical Approach, this study aimed to gain insight into the experiences and practices of SE’d workers as stories (narratives) pertinent to their life experiences and our research questions [62, 63]. This analysis was helpful for understanding how SE’d workers’ lives and experiences are embedded in broader social structures, such as neoliberal socio-economic structures. The analysis was composed of several phases: reviewing the transcripts multiple times, developing a codebook, establishing themes and subthemes, and identifying core narrative elements associated with each theme. A combination of deductive and inductive coding was used leading to a codebook of 10 codes, of which some were predetermined from the existing literature and research questions, and some were informed by issues identified during interviews. Using the qualitative data analysis software (NVIVO), the data sets were re-arranged in terms of the codebook. These codes helped us to reflect on the overall patterns of data, including descriptive themes. We then (re)viewed these descriptive themes and developed more analytical themes by grouping them together, moving back and forth between descriptive and analytical themes. This facilitated a higher level of abstraction and theorizing the interpretation of the research findings and the function they serve. As such, the narrative findings helped to show the experiences of SE’d workers under particular socio-economic structural conditions, including neoliberalism and precariat class structures. Reflecting on the lessons of neoliberalism [59] and precarity [20, 50, 51], our analysis resulted in the development of three key themes: anger, anomie, and anxiety. These are discussed below.

### Findings and discussion

As discussed above, SE’d workers form part of a precarious labor market [4, 20, 29, 46, 50, 51]. Our participants embodied neoliberalism-induced precarity within their life experiences of work, income, identity, and support systems. For example, a 26-year-old SE’d financial advisor, embodied his neoliberal agency strongly and enthusiastically during a discussion about his reasons for choosing SE. As he explained:

I wanted to be in control of my own time and my own freedom, right? And I want financial independence, so I don't like the salary. So that's why I think SE [does not] rely on government benefit too much, so they [those who want government benefits] had to understand that before you want to be self-dependent. The government is there but doesn't rely on it [rather advocating that everyone should have personal private insurance]. (Pablo)

A SE’d electrician similarly displayed his ‘entrepreneurial spirit’:

If you want to find something that's sustainable for you, for the long term and for your future family and generations to come, I don't see that in [regular] employment. I see the pride in being a business owner. (Paul)

This spirit fuelled his agency because he was not convinced that government could help or should have responsibility for SE’d people. In the view of this SE’d person, people ought to have considered SE pros and cons before engaging in this arrangement [64].

Such expressions by SE’d workers can be seen as the SE’d exploiting themselves, as has been elaborated by others in terms of labor (e.g., the commodification of labor), knowledge, and skills, and, more importantly, their deteriorating quality of life [52, 65]. Standing distinguished the exploitation of the precariat from the Marxist notion of the exploitation of proletariat within industrial capitalism by explaining how precariat workers exploit themselves, without being oppressed by an external bourgeoisie. For the precariat, according to Standing (2014), neoliberal state policy works as the bourgeoisie [11, 20, 51]. Although many participants in our study were lured into SE amid discourses of flexibility and freedom, most of them found that these freedoms were elusive. They found that business survival was difficult and often created excessive workloads that required juggling their business in addition to other employment. In this context, multiple jobs simultaneously performed by SE’d people exposes the high workload they had to maintain to stay afloat. Although regular employees often have multiple jobs, SE’d workers do this more often than salaried workers. According to Statistics Canada (2010), almost half of SE’d workers (who filed taxes) had income from other sources [66]. In this study, participants generally described having no vacations, holidays, or weekends, and they worked continually to keep their businesses afloat.

Standing (2014) posited that this class is exploited by itself; we extend this to suggest that government policy may work as a bourgeoisie counterpart. Accordingly, several SE’d participants expressed their concern about having excessive workloads, but were resigned to having a lifelong struggle to stay financially afloat. They were working hard to change their lot, to what extent they could.

This raises the question of who is responsible for such difficult circumstances. Is it the SE’d because they chose this type of work? This issue of choice needs to be revisited within the context of underlying systems and causes. In the disguise of autonomy, flexibility, and freedom, the SE’d are ‘free’ to work extra hours in a context that blurs distinctions between self- and super exploitation [44, 67–69]. Thus, flexibility is replaced with unpredictability and insecurity against the backdrop of current labor markets. In this context, a SE’d rotary commissioner, described how she had to work long hours and also look after her parents:

I do everything. I never hired employees; you know, I do my own advertising online. So, I don’t have a lot of time for myself to exercise or meditate. Like, I’m overweight right now; I’ve been overweight for a few years. … I get sick once in a while, right? I mean the normal cold, flu. […] Being self-employed, I am usually-… don’t finish work before seven. You know I am early because sometimes my clients call me early. So, I have very limited [free] time, you know -before starting work and after work. I look after my parents; I love the seniors. I don’t have any children, so you know I go to help them a lot. Yeah, I just have limited time for my house and my taking care of myself. (Ruby)

When their personal lives intersected with labour production, SE’d workers in our study possessed a “dual- burden” of vulnerabilities and marginalization. On the one hand, they were already caught in precariousness of SE. On the other hand, they became more vulnerable following any illness, injury, and related income loss or reduction. Precarious employment is increasingly understood as a critical social determinant of health, with this type of work having a pivotal impact on health and wellness [26, 28, 30, 52, 54, 70]. The adverse health effects of illness or injury are compounded by an absence of formal support systems that are accessible to the precariously employed [4, 23, 70, 71]. Thus, this process pushed many participants to the or non-standardized labor market, amid the prominent neoliberal tool of labour market ‘deregulation’ which, in turn, brings truncated social rights and inferior social status [20, 59].

Across the diversity of the SE’d participant experiences in this study were common experiences with respect to health and wellness. First, the SE’d participants experienced anger when their economic and social mobility was blocked by ill health [20]. For example, a SE’d information technology consultant had financial solvency, with annual earnings of $45,000. However, when he was required to be on bed rest for three months due to his leg injury, these unworked hours were not supported and he was unpaid. Due to the lack of income, to support his family he had to draw his savings, which were minimal, and on his wife’s income. In addition, his wife’s income was reduced by his illness because she had to miss work to provide him with care. This incurred substantial feelings of anger and anxiety regarding his life:

I had a big injury [..] a kind of spine injury […] I suffered a lot and it hampered me take away from my work [long discussion on his pain and sufferings] that has impacted me a lot in my income, […] for those unworked periods and that time it hampered me a lot […] felt helpless, not found meaning to live anymore.(Mamum)

Similarly, Ruby lived in a state of pain and financial scarcity, while continuing to work and being required to contribute to a federal pension plan. She felt anger and dissatisfaction about the mandatory (federal) pension contributions when she would rather use the money to support herself while unwell:

[…] have to pay whatever 3,4 or 5 thousand dollars, depending on what I made for pension. Which I say, I will only get if I survive to 65 because I don’t have kids or husband. It will go. You know, I talk to them about it once. I argued with them “What’s point of this. They [government] shouldn’t force me to save for pension, I can just do it for myself. And if I die before my 65, they get the money. I think it’s wrong there is no way around it.

These examples above illustrate how some of our participants felt unhappy and angry with their lives due to their low income and meagre social protections.

Second, the precariat encounter feelings of ‘anomie’ because they have to try continually to find meaningful work and income to stay afloat [20, 50]. They have few options but to exploit themselves in precarious employment, which brings physical and mental health repercussions [70]. In our study, a SE’d financial advisor encountered a volume of work that created a sleep disorder:

I think if I work too much, the stress, stress is bad, mental health, I guess ... Is it too much pressure? Sometimes I need time to relieve the pressure […] I have a terrible time sleeping. I have a sleep disorder […] So, of course, that affects daily life, right? When you don't have a good sleep, it's hard to function the next day. (Remi)

This SE’d worker attributed her poor quality of everyday life and vulnerabilities to her problematic work pattern:

You are isolated, and no one can talk to you. So, everyday life, quality of life is poor. Not eating, not sleeping, not living, not able to work, [just] make money and not yeah, just … very poor quality as life being, it’s yeah, I have to work every day, but you just have to rest (Remi)

Anomic conditions especially affected the following SE’d worker because she worried about her future health. Although she was healthy when interviewed, she saw that ill-health would bring difficulties because she could not afford income insurance:

Thank God because I’m healthy now, but I do worry about the future, I am getting older […] I’m a little bit worried and thinking I will be getting insurance in the future, but it so expensive the insurance too. […] I Don’t know what to do; I’m just hoping to stay healthy and managed, hopefully. (Ruby)

The participant ater mentioned that she could sell her car to cover expenses for her medications. Similarly, a SE’d DJ singer, Bob, had to sell his guitar due to lack of income during COVID-19. Such situations created circumstances and experiences for which they were not prepared, leading some SE’d workers in this study to express feelings of meaninglessness about life. Against a precarious backdrop, these workers had difficulty leading a decent life. In turn, such experiences might create public and population health risks, including chronic diseases and malnutrition.

A third aspect of the precariat was found in how many participants encountered elevated anxiety due to chronic financial insecurity and instability. Their feelings of precariousness escalated feelings of alienation due to a lack of social status and recognition by government [20]. For example, Remi encountered stress-induced sleep disorders that she saw as due to excessive work, and sleep disorders are potentially hazardous for health and related to anxiety [72, 73]. In this context, a SE’d cleaner, reflected that she had been suffering from elevated anxiety for several years, which had degraded her confidence and self-esteem:

The last few years, I hadn't realized previously that I do have some anxiety issues as well […] because of that reason, I didn't have a lot of confidence. […] Last year, actually my anxiety was really bad and everything. It was really bad. So, I actually put all my customers on hold, and I was going to take a few months off. (Sarika)

One SE’d participant, who suffered from a significant autoimmune disorder, stressed experiences of ‘dual diagnosis’ because she felt elevated stress and anxiety by thinking about income and survival, and this had adverse consequences on her wellness:

[…] because I couldn't afford a house […] my car got repossessed because my insurance claim had not kicked in yet. Ah, I, as a person who … has been chronically ill for a number of years […] its tight money. It's scary, and stress contributes to me being unwell […] I wouldn't deny the stress of trying to figure out how I'm going to pay for everything. … But it is a significant stressor, and if I ended up bedridden for three days -- because I was sick and that happened. I just make sure that I don't think about that because I don't have a choice. (Mary)

Although many participants described choosing SE voluntarily because they enjoyed flexibility and autonomy, these neoliberal ideas precariatized their social position at the expense of adequate living conditions and basic needs including food, shelter, and health. Importantly, this occurs within broader social structures of deregulation and privatization. Many participants accepted the financial and personal burden of risk associated with SE, leaving the state and government free of moral responsibility. Yet, several participants in our study described having no choice but to return to work following their illness or injury, even when still ill or impaired. For example, a gig worker started working within seven days of his hand injury, accepting the risk of further injury due to financial hardship. He had no other way to survive. As he noted:

Interestingly, the position of SE’d workers is inevitably unstable and ambiguous, day by day. This deintellectualization process has profound repercussions on young people because gig unemployment in particular is strategically compensated by promoting SE, which neoliberals justify by the urgent need to use labor resources flexibly. Regardless of the reasons behind this, this flexibility imposes heavy social costs for workers, expressed in financial loss or lower social status. The danger of lowering social status was a major cause of anxiety among SE’d workers in this study. For example, during the COVID-19 crisis, a SE’d actor felt overlooked when the government did not recognize the needs of SE’d workers: “I do think it is unfair [feeling anxiety due to identity crisis] and until Covid-19. It is like the government did not even notice that we are real people” (Jane). When COVID emergency benefits were later provided to SE’d workers, a SE’d data analyst felt hopeful that the government now recognized SE’d people: “I think it was good that I was recognized a little later than other people started to get their support and benefits” (Jimmy).

Overall, precarity had pivotal impacts on SE’d workers’ well-being by adversely psychologically affecting their personal, social and family lives with experiences of uncertainty and feelings of injustice, powerlessness, instability, and tension. This strain among the SE’d participants operated through high exposure to harmful physical and psychosocial work conditions; concern about the next contract extension; and social and material deprivation caused by poor income and under-protection, inability to engage in long-term life planning, such as family formation (e.g., delayed entry into marriage and having children) [68, 74–77]. Thus, through this pathway, precarious SE’d workers can fall into a cycle of deprivation, social exclusion, and marginalization, with limited upward social mobility [76].

## Conclusion and implications

There is limited research identifying solo-self-employed workers as belonging to a precariat class, and there is scant evidence revealing how their health and well-being are affected by work-related precarity. Using the lenses of political economy and critical sociology, and based on qualitative empirical data, this paper sought to fill this knowledge gap and contribute to an understanding of how post-industrial social structures, including neoliberalism, fabricate a class called ‘precariat’, entailing a social position that is vague, volatile, truncated, and contingent. Our findings highlight how people opt into SE because they prefer flexibility and autonomy in their working life. However, moving forward and in the guise of flexibility, they can encounter a life full of precarity including job unsustainability, unstable working hours and income, and exclusion from social benefits. As a result, the health and well-being of SE’d workers can be adversely affected by anger, anomie, and anxiety, bringing forward potential risks and threats for a growing population in the terrain of the future of work.

The findings in this research prompt the following policy implications and practices at the federal or provincial government level to better support the health and wellbeing of SE'd workers. Policy supports for today’s SE’d workers remain illusionary because they are based on a traditional picture of prosperous, entrepreneur who is not in need of state support. This policy approach is outdated in today’s context where many low-paying SE’d workers strive to lead a decent life. They face very difficult circumstances when they have to be away from work due to injury or sickness, as this strata of SE’d population generally cannot afford private insurance and lack access to many state supports geared to employees [78–80]. A recent Canadian study, based on 2016 census and tax data, revealed that gig workers rose from 5.5% in 2005 to 8.2% in 2021 [81]. According to the “2021 Canadian Self-employment Report”, nearly 7 million employees are expecting to make the jump to SE within the next two years, especially among those under the age of 35 years [81]. As such, a policy focus on young people who are SE’d may be needed. In all, for a sustainable sector of SE’d entrepreneurs, policy interventions may be needed to support SE'd people during periods of work disability or illness to help them to reintegrate into the workforce.

If SE’d workers are included in social security programs, it may create room for these workers to be covered by other programs, such as workers’ compensation, employment insurance, and employment standards. These changes would facilitate an equitable, inclusive, and sustainable social protection system, which is needed for a sustainable labour market, by facilitating labour market transitions and labour mobility.

There are several limitations to this research. First, in terms of recruitment, we recruited a good number of types of SE, but not all kinds of SE. However, we included many types, including gig workers (*n* = 3), information technology field (*n* = 2), art industry (*n* = 6), financial management (*n* = 3), tailoring, small business, electrician, construction worker, cleaner, rotary commissioner, catering, and home childcare provider. Second, we recruited more than half of the participants with a family income of 50 k and above [79]. As we recruited and conducted interviews online using digital platforms, we might not have reached a substantial number of low-income SE’d workers because they might have no access to digital technologies or recruiting platforms, such as Kijiji, and other social media might be out of their reach [79]. For example, we recruited only one participant who was a cleaner. Her experiences were different to those of other participants and enriched my data. More people from lower income groups could underpin my data. Third, as this was a student project, this study could not include people other than English speakers due to time and budget constraints [79]. Fourth, due to the COVID-19 Ontario provincial lockdown measure, we had to conduct audio/videoconferencing interviews. This created some practical challenges that conflicted with the holistic quality of qualitative research, including dropped calls, loss of intimacy, failure to capture the non-verbal communication and gestures, compared to in-person interviews. However, it provided a unique opportunity for the participants and the researchers by compressing the time–space divide, facilitating safety, reducing travel-related expenses, maintaining social distance, and protecting personal space and privacy [79]. Videoconferencing allowed this study to cover province-wide participants. Finally, we encountered much ‘absenteeism’ from some participants. For example, several people fixed an interview appointment, but ultimately, they did not appear for the interview. However, this issue is prevalent in the case of online interviews [82].

## Data Availability

The datasets used and/or analyzed during the current study are available from the corresponding author on reasonable request.
